# Sexual and Apogamous Species of Woodferns Show Different Protein and Phytohormone Profiles

**DOI:** 10.3389/fpls.2021.718932

**Published:** 2021-11-12

**Authors:** Helena Fernández, Jonas Grossmann, Valeria Gagliardini, Isabel Feito, Alejandro Rivera, Lucía Rodríguez, Luis G. Quintanilla, Víctor Quesada, Mª Jesús Cañal, Ueli Grossniklaus

**Affiliations:** ^1^Area of Plant Physiology, Department of Organisms and Systems Biology, Oviedo University, Oviedo, Spain; ^2^Functional Genomics Center, Zurich, Switzerland; ^3^Swiss Institute of Bioinformatics, Lausanne, Switzerland; ^4^Department of Plant and Microbial Biology & Zurich and Basel Plant Science Center, University of Zurich, Zurich, Switzerland; ^5^Servicio Regional de Investigación y Desarrollo Agroalimentario (SERIDA), Finca Experimental La Mata, Grado, Spain; ^6^Department of Biology and Geology, Physics and Inorganic Chemistry, Rey Juan Carlos University, Móstoles, Spain; ^7^Department of Biochemistry and Molecular Biology, Institute of Oncology of the Principality of Asturias, Oviedo University, Móstoles, Spain

**Keywords:** apogamy, apomixis, *Dryopteris affinis* ssp. *affinis*, *Dryopteris oreades*, fern, gametophyte, plant growth regulator, proteomic

## Abstract

The gametophyte of ferns reproduces either by sexual or asexual means. In the latter, apogamy represents a peculiar case of apomixis, in which an embryo is formed from somatic cells. A proteomic and physiological approach was applied to the apogamous fern *Dryopteris affinis* ssp. *affinis* and its sexual relative *D. oreades*. The proteomic analysis compared apogamous vs. female gametophytes, whereas the phytohormone study included, in addition to females, three apogamous stages (filamentous, spatulate, and cordate). The proteomic profiles revealed a total of 879 proteins and, after annotation, different regulation was found in 206 proteins of *D. affinis* and 166 of its sexual counterpart. The proteins upregulated in *D. affinis* are mostly associated to protein metabolism (including folding, transport, and proteolysis), ribosome biogenesis, gene expression and translation, while in the sexual counterpart, they account largely for starch and sucrose metabolism, generation of energy and photosynthesis. Likewise, ultra-performance liquid chromatography-tandem spectrometry (UHPLC-MS/MS) was used to assess the levels of indol-3-acetic acid (IAA); the cytokinins: 6-benzylaminopurine (BA), trans-Zeatine (Z), trans-Zeatin riboside (ZR), dyhidrozeatine (DHZ), dyhidrozeatin riboside (DHZR), isopentenyl adenine (iP), isopentenyl adenosine (iPR), abscisic acid (ABA), the gibberellins GA_3_ and GA_4_, salicylic acid (SA), and the brassinosteroids: brassinolide (BL) and castasterone (CS). IAA, the cytokinins Z, ZR, iPR, the gibberellin GA_4_, the brassinosteoids castasterone, and ABA accumulated more in the sexual gametophyte than in the apogamous one. When comparing the three apogamous stages, BA and SA peaked in filamentous, GA_3_ and BL in spatulate and DHRZ in cordate gametophytes. The results point to the existence of large metabolic differences between apogamous and sexual gametophytes, and invite to consider the fern gametophyte as a good experimental system to deepen our understanding of plant reproduction.

## Introduction

Understanding the factors underlying plant reproduction represents a challenge to which many research groups contribute given its enormous repercussions. In contrast to animals, in which the germ line differentiates early in development, flowering plants have no distinct germ line (Poethig et al., 1986). Instead, totipotent meristematic cells proceed through a long period of vegetative development before they eventually form complex sexual organs, the flowers (Vyskot and Hobza, 2004). Most angiosperms reproduce sexually through seeds, but the formation of seeds by asexual means is also possible, a process called apomixis. The production of seeds without sexual union is considered the holy grail of agriculture (Grossniklaus et al., 1998, 2001; Hofmann, 2010), but the molecular mechanisms operating behind apomixis remain unclear. Although plants with apomictic reproductive ability exceed 400 species (Grimanelli et al., 2003; Koltunow and Grossniklaus, 2003), no major seed crops are apomictic. Likewise, the distribution of apomictic taxa across plant lineages is uneven, with estimations of 0.1% in angiosperms, up to 10% in ferns, and with little or no evidence of its existence in gymnosperms, mosses, liverworts or hornworts (Lovis, 1978; Asker and Jerling, 1992; Dyer et al., 2012). Apomixis is especially frequent in the Dryopteridaceae family, which, together with Pteridaceae, comprises around 70% of the reported apomictic fern species (Liu et al., 2012).

Ferns represent a major vascular plant group, in which haploid and diploid generations are completely separated, and the gametophyte is an appealing experimental system to deal with reproduction. Moreover, in ferns, apomixis is an important mode of asexual reproduction, which has evolved several times independently within the group (Ekrt and Koutecký, 2016). Apomixis in ferns includes “apogamy,” the formation of sporophytes from somatic cells of the prothallium, and “agamospory” (or diplospory), which represents the production of unreduced (diplo) spores in the fronds. In contrast, in gametophytes reproducing sexually, there are two organogenic events, producing antheridia and archegonia, bearing the male and female gametes, respectively. Performing molecular analyses in ferns has been elusive, as they exhibit higher chromosome numbers and larger genomes than mosses and seed plants (Barker and Wolf, 2010), which made it difficult to obtain genomic data. However, the advent of next-generation sequencing (NGS) technologies, by which it is possible to characterize the transcriptome in plants, represents a small but information reach-target compared to complete genome characterization (Ward et al., 2012). The variation in gene expression, induced by whatever environmental or endogenous conditions, can be examined in non-model organisms because these techniques have become more feasible as automation and efficiency have reduced costs. Until present, some transcriptome and proteome data sets have been published for ferns, which include the species *Pteridium aquilinum* (Der et al., 2011), *Ceratopteris richardii* (Salmi et al., 2010; Cordle et al., 2012), *Blechnum spicant* (Valledor et al., 2014), *Lygodium japonicum* (Aya et al., 2015), and *D. affinis* ssp. *affinis* (Grossmann et al., 2017; Wyder et al., 2020). Over the last case, both transcriptomic and proteomic analyses were performed by using next-generation sequencing (NGS) and shotgun proteomics by tandem mass spectrometry (MS/MS).

Several papers have been published to deepen on sexual and asexual reproduction in ferns. Two of them were driven in the model fern species *C. richardii*, looking for genes associated with female expression, and also male expression mediated by the pheromone antheridiogen in gametophytes (Atallah et al., 2018; Chen et al., 2019). Also, a comparative transcriptome analyses was done by Fu and Chen (2019) between apogamous and sexual gametophytes in *Adiantum reniforme* var. *sinense*. Previously, Bui et al. (2017) in *C. richardii* reported an AINTEGUMENTA-LIKE unigene, inducing the sporophyte formation without fertilization. It mirrors *BABY BOOM* (*BBM*) gene, a transcription factor of AP2/ERF family, which in angiosperms are known to promote somatic embryogenesis. Likewise, Domzalska et al. (2017) found proteins differently regulated during somatic embryogenesis in the tree fern *Cyathea delgadii*. Freshly, in the apogamous species, we have done a RNA-Seq approach to compare gene expression profiles of one- and two-dimensional gametophytes, finding several thousands of genes differentially expressed, and related to different aspect of either vegetative or reproductive behavior of the gametophyte (Wyder et al., 2020). In summary, with the increasing availability of genomic data from non-model species, better approaches will improve the sensitivity in protein identification for species distantly related to models.

On the other hand, phytohormones take part in the regulation of almost all phases of plant development, and also mediate the responses to various environmental stresses (Hicks, 1894; Rademacher, 2000; Dobrev et al., 2002). In ferns, growth and development of gametophyte and sporophyte are controlled by plant growth regulators, receiving especial attention their applications on morphogenesis, and its great repercussion on the sporophyte multiplication of ornamental ferns (Amaki and Higuchi, 1992; Fernández and Revilla, 2003; Somer et al., 2010; Rybczynski et al., 2018; Singh and Johari, 2018). The impact of phytohormones on gametophyte reproduction has been scrutinized in several species, some of them governed by an antheridiogen system, such as *B. spicant*, being these compounds devoted to promote genetic exchange, and linked chemically to gibberellin-related diterpenoids (Yamane, 1998; Menéndez et al., 2006a,b; Kazmierczak, 2010; Tanaka et al., 2014; Valledor et al., 2014). In *B. spicant*, Menéndez et al. (2006b) found similar levels of the gibberellins GA_4_, GA_7_, and GA_20_ in male and female gametophytes, while ABA has been reported to act as an antheridiogen antagonist in *C. richardii* (Warne and Hickok, 1989). In the sexual fern *Asplenium nidus*, which lacks antheridiogens, a significant increase in the content of the cytokinins iP and iPR was found in female gametophytes, and also qualitative differences in the content of gibberellins between the gametophyte and sporophyte generations were reported (Menéndez et al., 2011). In the gametophytes of *Polystichum aculeatum* and *Dryopteris filix-mas*, qualitative and quantitative changes of phytohormone content associated with the regulation of growth and gametangia formation, were recently documented (Kosakivska et al., 2019, 2020). On the other hand, variations in the content of phytohormones have been noted in *D. affinis* ssp. *affinis* (Menéndez et al., 2006c), associated to apogamy process. Yet, little attention has been paid so far to other phytohormones such as SA or brassinosteroids in basal branching vascular plants (i.e., lycophytes and ferns) (Sun et al., 2010; Choudhary et al., 2012). The former was traditionally associated with pathogen defense, together with jasmonic acid (de Vries et al., 2018), and the latter, in junction with auxins and gibberellins, is part of a key subset of plant hormones considered major determinants of plant growth and development (Ross and Reid, 2010; Gómez-Garay et al., 2018).

For a better understanding of the functions of the phytohormones and even possible interactions between them, exhaustive determination of their contents is of great importance (Bai et al., 2010). Analyses of phytohormones have focused on auxins, gibberellins, cytokinins, abscisic acid, or ethylene, while others like jasmonates or brassinosteroids were recently added, as standards and protocols became available. In the last few years, the advent of rapid, sensitive, accurate and efficient methods, even when small amounts of sample are available, has made possible to broaden the number of compounds analyzed (Du F. et al., 2012; Porfírio et al., 2016; Delatorre et al., 2017). HPLC-MS is the most accurate method to perform quantitative analysis of endogenous phytohormones or plant regulators (Pan et al., 2008, 2010; Delatorre et al., 2017), and at present day, a step forward is the use of UHPLC to detect those small amounts of phytohormones in fresh tissue, giving even better results on a wide spectrum of plant hormones.

In this work, a physiological and proteomic study was carried out in the fern *D. oreades* and its close relative *D. affinis* ssp. *affinis* (hereafter referred to as *D. affinis*). *D. oreades* is a sexual diploid and thus its gametophytes are haploid. *D. affinis* is also diploid but has two distinct genomes, one from *D. oreades* and one from an unknown “pure” *D. affinis* ancestor (Fraser-Jenkins, 1980). *D. affinis* is obligately apogamous and its gametophytes are diploid with the same genome constitution as that of sporophytes (Sheffield et al., 1983). Thus, this species pair provides an experimental system for investigating the gene expression an phytohormonal changes linked to sexual or apogamous reproduction. Specifically, our analyses involved (1) a proteomic comparison between sexual and apogamous gametophytes, and (2) the assessment of the endogenous content of fourteen phytohormones in sexual gametophytes and in three developmental stages of apogamous gametophytes: one-dimensional or filamentous; and two-dimensional or spatulate, and heart-shaped or cordate.

## Materials and Methods

### Plant Material and Growth Conditions

Spores of *D. affinis* were obtained from sporophytes growing in Turón valley (Asturias, Spain), 477 m a.s.l., 43°12′10″N−5°43′43″W. In the case of *D. oreades*, spores were collected from sporophytes growing in Burgos, Neila lagoons, 1920 m a.s.l., 42°02′48″N−3°03′44″W. Spores were released from sporangia, soaked in water for 2 h, and then washed for 10 min with a solution of NaClO (0.5%) and Tween 20 (0.1%). Then, they were rinsed three times with sterile, distilled water. Spores were centrifuged at 1,300 *g* for 3 min between rinses, and then cultured in 500-mL Erlenmeyer flasks containing 100 mL of liquid Murashige and Skoog (MS) medium (Murashige and Skoog, 1962). Unless otherwise noted, media were supplemented with 2% sucrose (w/v), and the pH adjusted to 5.7 with 1 or 0.1 M NaOH. The cultures were kept at 25°C under cool-white fluorescent light (70 μmol m^−2^s^−1^) with a 16:8 h light: dark photoperiod, and put on an orbital shaker (75 rpm).

**Figure 1:**
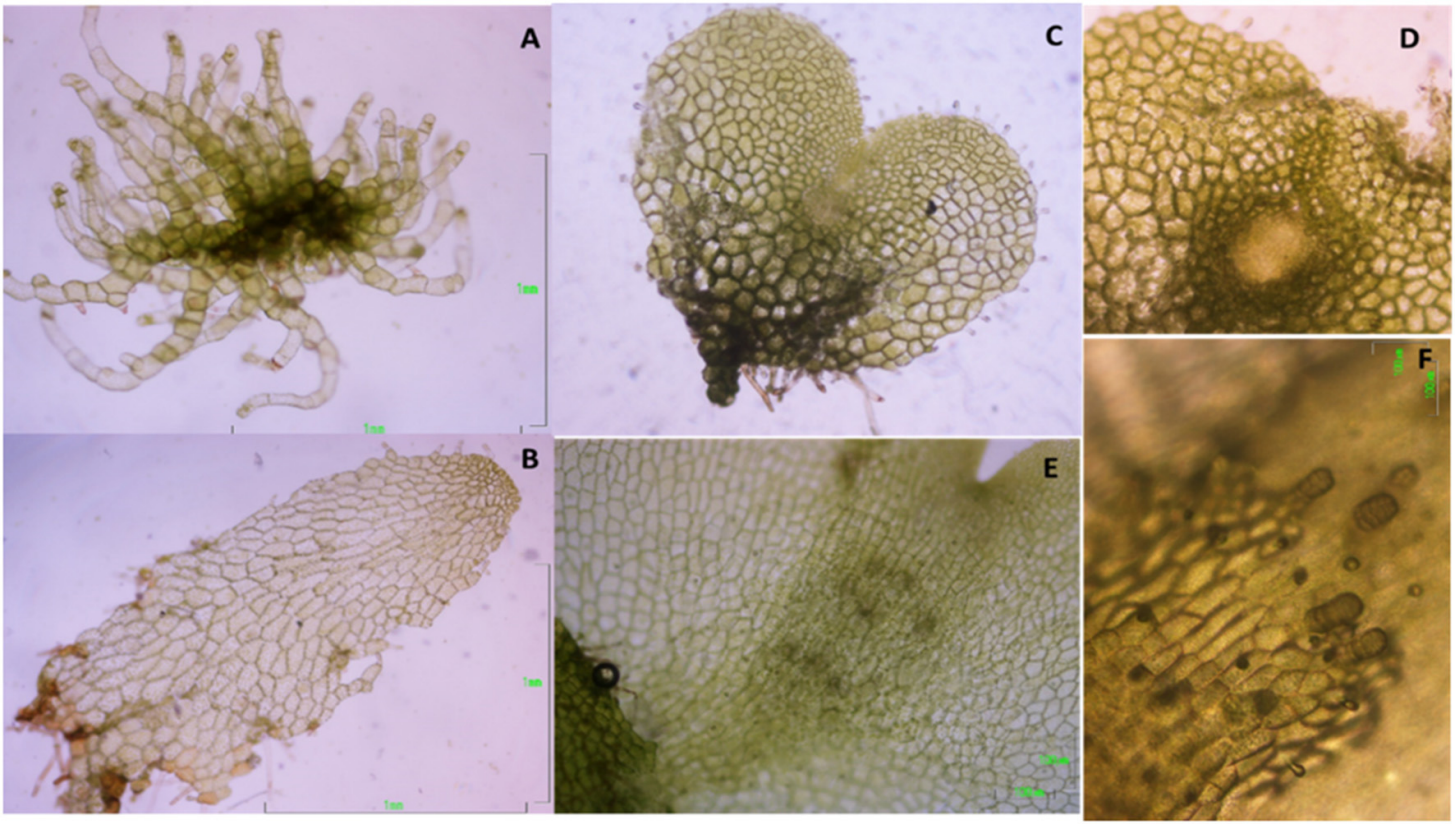
Morphological features in the growth stages of the apogamous fern *Dryopteris affinis*: **(A)** filamentous; **(B)** spatulate; **(C)** cordate or heart-shaped; **(D)** detail of first steps in the embryo development; and its sexual relative *D. oreades*: **(E)** archegonium cushion in the middle of gametophyte; **(F)** archegonia.

Following spore germination, gametophytes of both species go through three sequential growth stages, as in most ferns (Nayar and Kaur, 1971): filamentous, because of one-dimensional growth; spatulate, when growth becomes two-dimensional; and cordate (heath-shaped), when a notch meristem is formed in the middle of the upper margin. Gametophytes of *D. affinis* were collected at these three stages (Figures 1A–C), and, in the case of cordate ones, with visible signs (under a light microscope) of an evolving apogamic center (Figure 1D), composed of smaller and darker isodiametric cells. Filamentous gametophytes were collected 30 days after the start of spore culture. Spatulate and cordate gametophytes were obtained by transferring 30-day old filamentous gametophytes to 200 mL flask containing 25 mL of MS medium supplemented with 2% sucrose (w/v) and 0.7% agar, and being collected after 20 or 30 additional days, respectively. Gametophytes of *D. oreades* needed around 6 months to become cordate and reach sexual maturity (Figures 1E,F). They were then picked up and, in all cases, only female reproductive organs (i.e., archegonia) were observed under a light microscope. Samples of the three types of apogamous gametophytes (filamentous, spatulate and cordate) and sexual cordate, were weighted before and after being lyophilized for 48 h (Telstar-Cryodos) and stored in *Eppendorf* tubes on a freezer at −80°C until required.

### Protein Extraction

From the cordate apogamous and cordate sexual gametophytes (three samples each), an amount of 20 mg dry weight of gametophytes were homogenized using a Silamat S5 shaker (Ivoclar Vivadent, Schaan, Liechtenstein). Homogenized samples were solubilized in 800 μL of buffer A [0.5 M Tris-HCL pH 8.0, 5 mM EDTA, 0.1 M Hepes-KOH, 4 mM DTT, 15 mM EGTA, 1 mM PMSF, 0.5% PVP and 1 × protease inhibitor cocktail (Roche, Rotkreuz, Switzerland)] using a Potter homogenizer (Thermo Fisher Scientific, Bremen, Germany).

Proteins were extracted in two steps: first, the homogenate was subjected to centrifugation at 16,200 *g* for 10 min at 4°C on a table top centrifuge and, second, the supernatant was subjected to ultracentrifugation at 117–124 kPa (~100,000 *g*) for 45 min at 4°C. Post-ultracentrifugation the supernatant contained the soluble protein fraction. The pellet from the first ultracentrifugation was re-dissolved in 200 μL of buffer B (40 mM Tris base, 40 mM DTT, 4% SDS, 1 × protease inhibitor cocktail (Roche, Rotkreuz, Switzerland) to extract membrane proteins using ultracentrifugation as described before. The supernatant after the second ultracentrifugation step contained the membrane protein fraction. Ultracentrifugation was performed using an Airfuge (Beckman Coulter, Pasadena, CA).

Protein concentrations were determined using a Qubit Fluorometer (Invitrogen, Carlsbad, CA). For 1 D gel electrophoresis, ~1 mg protein per each soluble and membrane fraction was loaded separately onto a 0.75 mm tick, 12% SDS-PAGE mini-gel. Samples were treated with sample loading buffer and 2 M DTT, heated at 99°C for 5 min, followed by a short cooling period on ice, and loaded onto the gel. 1D gel electrophoresis was performed at 150 V and 250 mA for 1 h in 1X Running Buffer.

### Protein Separation and In-gel Digestion

After 1D SDS-PAGE each gel lane was cut into six 0.4 cm wide sections using a custom-made gel cutter, resulting in 48 slices. These slices were further fragmented into smaller pieces and subjected to 10 mM DTT (in 25 mM AmBic pH8) for 45 min at 56°C and 50 mM Iodoacetamide for 1 h at room temperature in the dark, prior to trypsin digestion at 37°C overnight (Baerenfaller et al., 2008). The small pieces were washed twice with 100 μL of 100 mM NH_4_HCO_3_/50% acetonitrile, and washed once with 50 μL acetonitrile. All three supernatants were discarded and peptides digested with 20 μL trypsin (5 ng/μL in 10 mM Tris/2 mM CaCl_2_, pH 8.2) and 50 μL buffer (10 mM Tris/2 mM CaCl_2_, pH 8.2). After microwave-heating for 30 min at 60°C, the supernatant was removed and gel pieces extracted once with 150 μL 0.1% TFA/50% acetonitrile. All supernatants were combined and dried, and samples were then dissolved in 15 μL 0.1% formic acid/3% acetonitrile and transferred to auto-sampler vials for liquid chromatography (LC)-MS/MS where 5 μL were injected.

### Protein Identification, Verification, and Bioinformatic Downstream Analyses

MS/MS and peptide identification (Orbitrap XL) were performed accordingly (Grossmann et al., 2017). Scaffold software (version Scaffold 4.2.1, Proteome Software Inc., Portland, OR) was used to validate MS/MS-based peptide and protein identifications. Mascot results were analyzed together using the MudPIT option. Peptide identifications were accepted if they scored better than 95.0% probability as specified by the Peptide Prophet algorithm with delta mass correction, and protein identifications were accepted if the Protein Prophet probability was above 95%. Proteins that contained same peptides and could not be differentiated based on MS/MS alone were grouped to satisfy the principles of parsimony using scaffolds cluster analysis option. Only proteins that met the above criteria were considered positively identified for further analysis. The amount of random matches was evaluated by performing the Mascot searches against a database containing decoy entries and checking how many decoy entries (proteins or peptides) passed the applied quality filters. The peptide FDR and protein FDR was estimated at 2 and 1%, respectively, indicating the stringency of the analyses.

A semi-quantitative spectrum counting analysis was conducted. The “total spectrum count” for each protein and each sample was reported, and these spectrum counts were averaged for each species, *D. affinis* (DA) and *D. oreades* (DO). Then, one “1” was added to each average (DA and DO) in order to prevent division by zero and then a log2-ratio of the averaged spectral counts from DA vs. DO was calculated. Proteins were considered to be differentially expressed if this log2Ratio is above 0.99. This refers to at least twice as much peptide spectrum match (PSM) assignments in one group compared to the other. Also, in order to have a functional understanding of the identified proteins, we blasted the whole protein sequences of all identified proteins against *Sellginella moellendorfii* and *Arabidopsis thaliana* Uniprot sequences and retrieved the best matching identifier from each of them, along with the corresponding e-value, accepting blast-hits which evaluate below 1E-7. These better described ortholog identifiers are then used in further downstream analysis.

### Protein Analysis Using STRING-DB Platform

The identifiers of the genes from the apogamous and sexual gametophyte samples were used as input for String version 11.0 analysis and networks were built based on high confidence in the ranking (0.7).

### Endogenous Phytohormone Analyses

The extraction of phytohomones was done from 0.2 g fresh weight of each of the four gametophyte samples (filamentous, spatulate and cordate apogamous; and cordate sexual), according to the protocol of Pan et al. (2008) with modifications from Delatorre et al. (2017), which avoid derivatization and purification of the samples, and six replicates were considered for sample. The phytohormones analyzed were: ABA, IAA, the cytokinins iP, iPR, Z, ZR, DHZ, DHZR, the gibberellins GA_3_ and GA_4_, SA, and the brassinosteroids BL and CS (Sigma-Aldrich, St. Louis, MO, USA). The following deuterated standards were employed: IAA-d_5_, SA-d_6_, DHZ-d_3_, and ABA-d_6_ (20 ng); GA_9_-d_2_ and CS-d_5_ (40 ng) and BA-d_7_ (10 ng). All of them were supplied by Olchemim Ltd. (Czech Republic) except d6-SA obtained from Sigma Aldrich (St. Louis, MO, USA). Starting from these internal standards, known samples homogenized in liquid N_2_, were extracted with 1 ml of extraction buffer (2-propanol/Mili Q water/Hydrochloric acid; 2:1:0.002; v/v/v) in 2 mL propylene Eppendorf tubes (Eppendorf Safe Lock England), and agitated by repeated inversion (60 rpm) for 20 min, at 4°C, in the dark. The resulting homogenate was transferred to a teflon tube (Oak Rifge Centrifuge Tube, Thermo Scientific, England) with 1.8 ml dichloromethane and reextracted by repeated and inverted agitation for 30 min, at 4°C in the dark. After 30 min standing, three phases were obtained, an aqueous, a material debris and an organic phase. The organic bottom phase was recovered and the intermediate debris phase was also re-extracted again. The propanol of combined extracts was evaporated under N_2_ flow. Resulting dried extracts were resuspended in 150 μL of 100% methanol and filtered through a 0.2 μm regenerated cellulose Captiva Premium Syringe filter (Agilent Technologies, California, USA). The chromatographic separation takes place on a reverse phase chromatographic column (*Zorbax* SB-C18 2.1 × 50 mm) coupled to a *Zorbax* plus eclipse pre-column (C18 2.1 × 5 mm) at 40°C. Two solvents are used as mobile phases with a flow rate of 0.45 mL / min: MeOH acidified with 0.1% formic acid (solvent A) and ammonium formate 10 mM, pH 4 (solvent B).

Samples were injected into UHPLC System (1290 Infinity binary LC system, Agilent Technologies, Madrid Spain). The UHPLC was coupled to a Triple Quadrupole (6460 Triple Quad, LC/MS equipped with ESI-Ion Source). All compounds were separated and quantified following the protocol described by Delatorre et al. (2017).

### Statistical Analyses of Phytohormone Content

Deviation from normality and homogeneity of variance were tested respectively with Shapiro-Wilk and Levene tests. One-way ANOVA was carried out with a Tukey-HSD test to group samples. From the obtained means squares estimates, values were used to identify the role played by each kind of gametophyte in overall variability through the Principal Component Analysis (PCA). This PCA was performed on the mean values recorded for 14 phytohormones assessed from four kind of gametophytes. From this PCA, a bioplot graph was drawn in an attempt to classify the endogenous content of phytohormones into similarity groups. All statistical analyses were completed in R environment with R Studio (Team, 2016) or the free software from *Past.uio 3.26* (Hammer, 2001).

## Results

### Identification of Differentially Expressed Genes by LC-MS/MS

**Figure 2:**
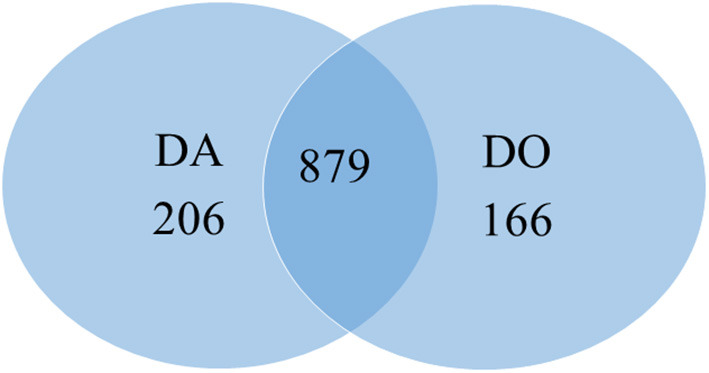
Venn diagram totaling the number of proteins similarly and differently regulated in heart-shaped gametophytes from the apomictic fern *Dryopteris affinis* (DA), and its sexual relative *D. oreades* (DO).

**Figure 3:**
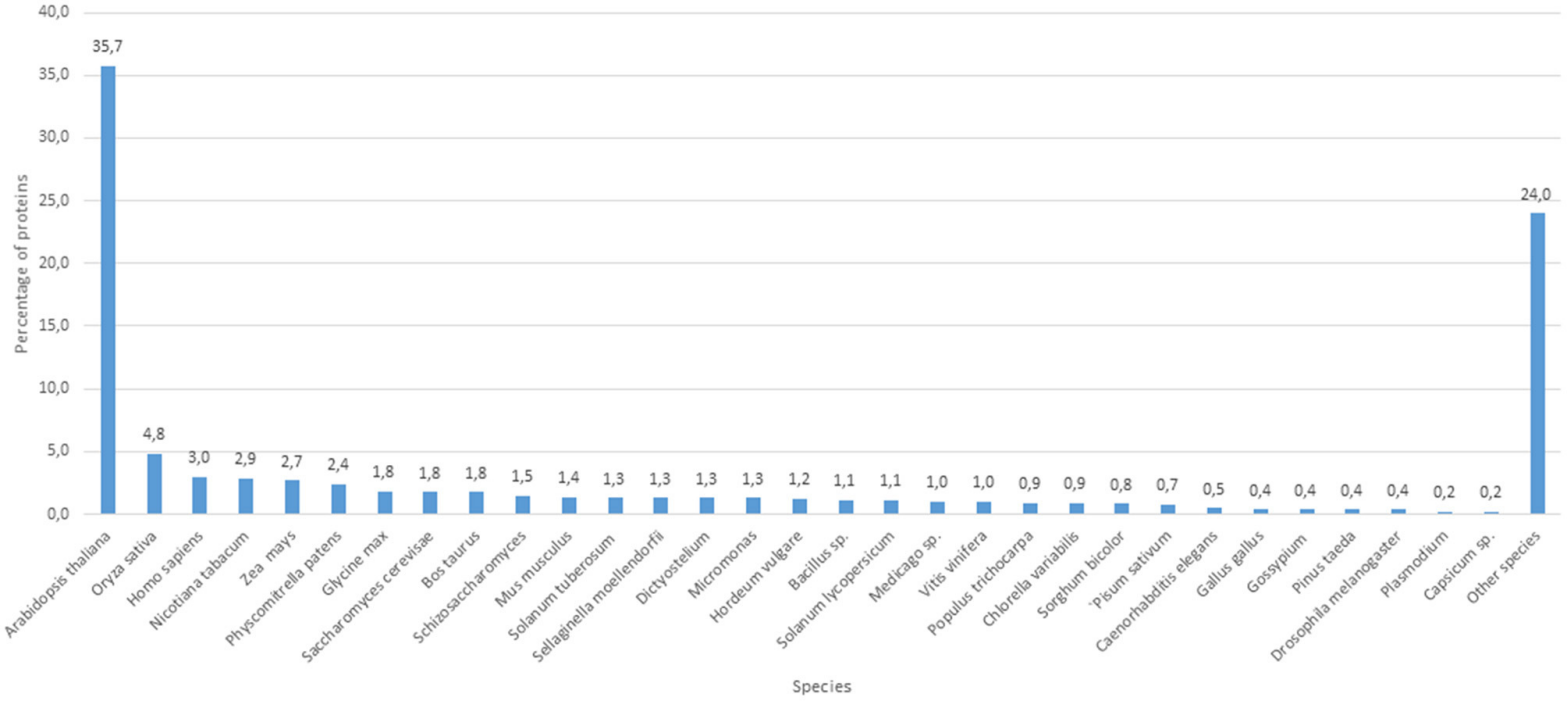
Number of protein clusters obtained from the apogamous gametophytes of *Dryopteris affinis* and its sexual relative *D. oreades*, with the best hits to species belonging to the phylogenetic groups specified.

The proteomic analyses generated a total of 879 quantifiable proteins (Supplementary Table 1), rejecting those proteins not having a homolog and those having two DA/DO proteins the same homolog and repetitions. The “differential” protein regulation between the apogamous species and its sexual relative took into account proteins that were not “borderline,” exhibiting a total of three assigned spectra, and rejecting those with less counts. According to this criterium, from the total number of annotated proteins, 206 and 166 were upregulated in 2 month-old apogamictic and sexual gametophytes, respectively (Figure 2). One-third of total annotated proteins (35.7%) had BLAST hits to proteins from *A. thaliana*, followed by *Oryza sativa* (4.7%). The remain matches of proteins are distributed among several species such as the seed plants *Nicotiana tabacum, Zea mays, Glycine max, Solanum tuberosum*, etc., animals like *Bos taurus, Mus musculus, Caenorhabditis elegans*, seedless plants like the moss *Physcomitrella patens* or the lycophyte *Sellaginella moellemdorfii*, and microorganisms such as *Saccharomyces cerevisiae, Bacillus* sp., etc. (Figure 3).

### Over-represented Categories for Differentially Regulated Proteins in Apogamous and Its Sexual Relative Gametophyte

The collections of differentially regulated proteins were analyzed by using the STRING database (http://String-db.org). Regarding those of the apogamous species, the results show 206 nodes and 1,047 edges, and a PPI enrichment *p*-value = 1.0 e^−16^; in the sexual relative, 166 nodes and 259 edges were scored, with a PPI enrichment *p*-value = 5.55 e^−16^. In both of cases, data reflected that our proteins have more interactions among themselves than what would be expected for a random set of proteins of similar size, drawn from the genome. Thus, the proteins are at least partially biologically connected, as a group.

**Figure 4:**
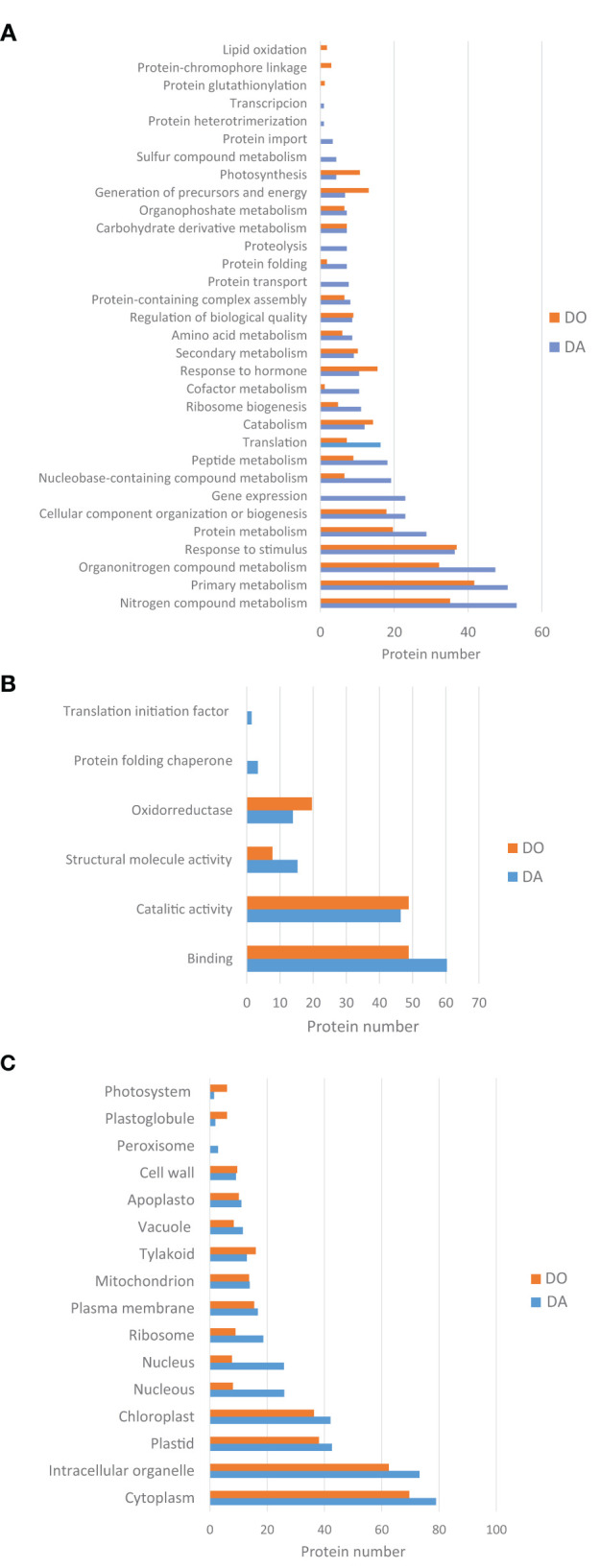
GO classification of proteins differentially upregulated in the apogamous fern *Dryopteris affinis* and its sexual relative *D. oreades*, in the three main categories: **(A)** biological function; **(B)** molecular function; and **(C)** cellular component.

In order to obtain a better knowledge about the function of both collections of proteins, Gene Ontology (GO), and KEGG enrichment analyses provided by the String platform were obtained. In total, 206 and 166 proteins differentially regulated were clustered to a number of GO and KEGG pathways significantly enriched (FDR's). Regarding the GO databank, proteins were checked according to standard categories of biological function, molecular function and cellular components (Figure 4). In both species, more than half of differentially regulated proteins (60–70%) counted for general metabolic and cellular processes (Figure 4A). The apogamous species, totals more proteins involved in the primary metabolism like amino acids, peptides and proteins (including folding, transport and proteolysis), nucleic acids and cofactors, nitrogen and sulfur metabolism, ribosome biogenesis, gene expression and translation. Instead, the sexual counterpart accumulated more proteins involved in generation of precursor metabolites and energy, and photosynthesis. In addition, it was found more proteins involved in response to hormones in this species. There are also biological functions such as response to stimulus or metabolism linked to carbohydrates, in which both species show a similar increase in the number of proteins scored but different profiles when detailing each kind of processes.

Under “molecular function” category, the list of proteins differentially regulated was headed by binding activity in both species, being a ten percent higher in the apogamous gametophyte (Figure 4B). This activity is dominated by organic cyclic compounds and ion binding, in both gametophytes, respectively, followed by oxidoreductase activity, higher in the sexual gametophyte. The apogamous gametophytes expressed more proteins related to structural molecule activity, protein folding chaperone and translation initiation factor.

Additionally, under “cellular components” most part of the proteins was linked to cytoplasm and intracellular organelles, especially plastids and chroloplasts (Figure 4C). Both species upregulated proteins involved in cell wall, plasma membrane, and mitochondrion. In the apogamous gametophytes, it was significant the presence of proteins linked to nucleus, ribosome and peroxisome. On the other hand, in the sexual relative, increased the content of proteins associated to the photosynthetic machinery such as tylakoids, plastoglobule, and photosystems.

**Figure 5:**
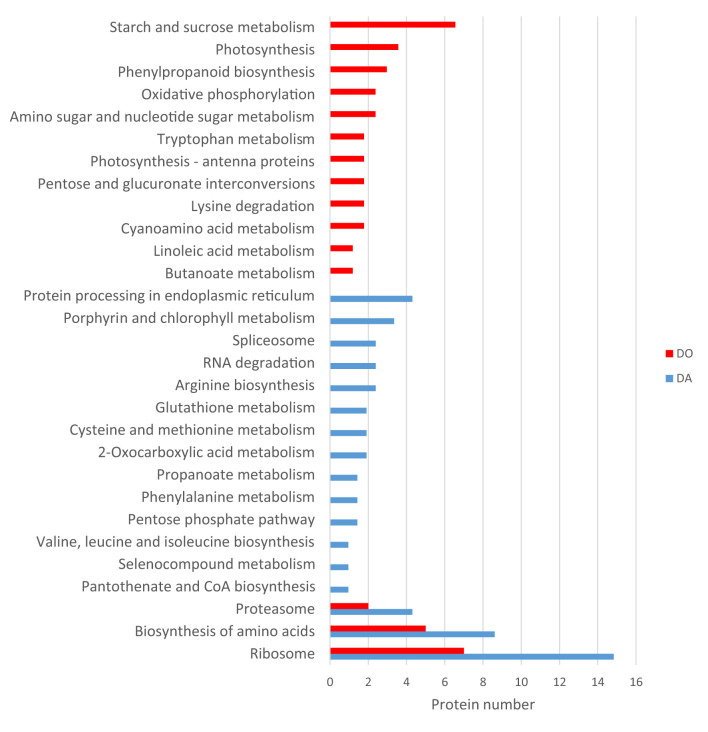
KEGG classification of proteins differentially upregulated in the apogamous fern *Dryopteris affinis* and its sexual relative *D. oreades*.

KEGG clustering showed an important expression of proteins associated to ribosome, aminoacid metabolism and proteasome in both species, being higher in the apogamous gametophyte (Figure 5). In this species, proteins drop in protein processing in endoplasmic reticulum, porphyrin and chlorophyll metabolism, spliceosome, RNA degradation, arginine biosynthesis, etc. In the sexual counterpart, proteins are involved in starch and sucrose metabolism, amino sugar and nucleotide sugar metabolism, photosynthesis, oxidative phosphorylation, lipid metabolism, etc. A selection of those proteins differentially regulated is reported in Tables 1, 2.

**Table 1:** Selected proteins upregulated in the apogamous fern *Dryopteris affinis* ssp. *affinis*.

**Description**	**Idex in Supp table**	**Accession number**	**MW kD**	**Uniprot accessions**	**e-Evalue**	**log2Ratio_DAvsDO**	**% Coverage**	**Total unique peptide count**
GTP-binding nuclear protein Ran-3	325	43286-387_3_ORF1 (+68)	29	Q8H156	1.8912E-157	1.00	14	6
Puromycin-sensitive aminopeptidase	1,071	166554-171_3_ORF1 [7]	115	F4I3R1	0	2.32	2.5	6
Metallopeptidase M24 family protein	589	207675-131_6_ORF2	43	Q96327	0	1.32	4.9	4
40S ribosomal protein S6-2	332	7438-849_3_ORF2	29	P51430	1.1336E-150	1.50	24	9
DEAD-box ATP-dependent RNA helicase 3	987	133309-212_3_ORF2 [8]	84	Q8L7S8	0	2.13	13	13
Maternal effect embryo arrest 59 (MEE59)	304	102286-246_3_ORF2	28	O23157	4.92172E-18	2.32	7.5	2
Chromo domain-containing protein LHP1	828	152641-192_2_ORF2 (+1)	59	Q946J8	1.00311E-43	3.17	10	5
Transducin/WD40 repeat-like superfamily protein	182	362879-38_5_ORF2 (+2)	22	Q9SZQ5	1.5458E-164	1.42	2	3
AtLa1	720	278034-81_6_ORF2	50	Q93ZV7	1.05674E-96	1.74	12	5
Embryo defective 1473 (emb1473)	336	188230-148_1_ORF2 (+1)	30	Q9SYL9	4.9526E-104	1.42	10	4
Methyl-CpG-binding domain-containing protein 10	435	254886-96_1_ORF2 (+1)	34	Q9XI36	1.22143E-24	2.32	8.9	2
Pentatricopeptide repeat-containing protein At1g63130	1,010	62277-321_4_ORF2	90	Q9CAN0	3.09193E-32	2.00	5.1	7
D-3-phosphoglycerate dehydrogenase 1	918	229414-114_3_ORF1	68	O49485	0	1.00	23	14
DC1 domain-containing protein	919	167954-169_4_ORF1	68	O80763	8.1609E-154	1.58	5.4	3
Dihydrolipoyl dehydrogenase 1	867	143453-204_6_ORF2 [2]	63	A8MS68	0	1.58	13	9
Membrane-associated progesterone binding protein 2	65	142203-205_1_ORF1	15	Q9SK39	1.60057E-42	1.58	19	3
Magnesium-chelatase subunit ChlD	985	3146-1200_2_ORF1	84	Q9SJE1	1.5299E-178	1.32	4.5	3
Magnesium-chelatase subunit ChlH	1,103	102670-246_5_ORF2 (+4)	159	Q9FNB0	0	1.00	1.9	2
P-loop containing nucleoside triphosphate hydrolases superfamily protein	718	237628-108_3_ORF2 (+2)	50	P16127	0	1.58	14	7
Coproporphyrinogen-III oxidase 1	639	263607-90_6_ORF2 (+1)	46	Q9LR75	0	3.00	11	3
Carbamoyl-phosphate synthase large chain	1,088	39629-404_3_ORF2 [3]	133	Q42601	0	1.32	2.6	3
Argininosuccinate synthase	740	1466-1535_4_ORF1 [4]	52	Q9SZX3	0	1.00	7.1	3
Prohibitin-3	372	25450-498_6_ORF2 [5]	31	O04331	1.6594E-147	1.00	15	5
Acetyl-CoA carboxylase 1	1,122	1892-1420_1_ORF2 [3]	265	Q38970	0	1.22	12	28
Nucleoid-associated protein At2g24020	145	16884-601_6_ORF1	20	O82230	7.88432E-68	1.00	11	2
Winged-helix DNA-binding transcription factor family protein	646	275958-82_2_ORF1	46	F4INW2	4.61845E-21	2.58	16	8
60S acidic ribosomal protein family	12	63281-318_4_ORF1	9	Q9FLV1	2.36329E-27	3.00	51	2
Eukaryotic translation initiation factor 3 subunit C	1,068	205951-132_1_ORF2 (+3)	113	O49160	0	2.32	6.5	7
Triosephosphate isomerase	441	151710-194_2_ORF2 [4]	34	Q9SKP6	1.3778E-154	2.22	6.9	8
Lactate/malate dehydrogenase family protein	717	130359-215_1_ORF1	50	Q8H1E2	0	1.87	16	7
Homolog of human CAND1	1,090	112593-234_6_ORF2	137	Q8L5Y6	0	2.32	2.6	3

**Table 2:** Selected proteins upregulated in the sexual fern *Dryopteris oreades*.

**Description**	**Idx in spp table**	**Accession number**	**MW (kD)**	**Uniprot accessions**	**Evalue**	**log2Ratio _DAvsDO**	**% Coverage**	**Total unique peptide count**
Alpha-amylase-like	827	266991-88_5_ORF2	59	Q8VZ56	7.9347E-154	−1	7.1	3
Beta-amylase 2	857	190175-146_3_ORF2 (+1)	62	O65258	0	−2.81	6	4
pfkB-like carbohydrate kinase family protein	621	222710-119_2_ORF2 [5]	45	Q9C524	2.947E-180	−1.66	19	9
Phosphoglucan, water dikinases	1,089	316777-59_1_ORF2	135	Q6ZY51	0	−2.08	18	17
Probable sucrose-phosphate synthase 3	1,081	165303-173_5_ORF1 [19]	123	Q8RY24	0	−2.81	7.3	9
Glucose-1-phosphate adenylyltransferase small subunit	809	tr|A9SGH8|A9SGH8_PHYPA	57	P55228	0	−1.22	14	6
Glucose-6-phosphate isomerase 1	933	373594-34_6_ORF1 [7]	70	Q8H103	0	−1	13	6
Fructose-1,6-bisphosphatasa	491	tr|D8RRD1|D8RRD1_SELML	37	Q9MA79	0	−2.32	12	6
Linoleate 9S-lipoxygenase 1	1,041	38467-410_3_ORF2	102	Q06327	0	−1.58	3.8	3
Lipoxygenase 3	1,027	61725-322_6_ORF3	95	Q9LNR3	0	−3	8.5	8
Peroxisomal fatty acid beta-oxidation multifunctional protein MFP2	977	46601-373_3_ORF2	80	Q9ZPI5	0	−1.32	3.2	3
Monocopper oxidase-like protein SKU5	895	149426-198_2_ORF2	65	Q9SU40	0	−1.42	13	6
Late embryogenesis abundant protein (LEA) family protein	184	415079-17_4_ORF2 (+1)	22	Q9SUB2	0.000692152	−1.07	31	7
Late embryogenesis abundant protein 31	376	43963-384_3_ORF2 [2]	31	Q9LJ97	4.74152E-40	−1.15	30	8
Late embryogenesis abundant protein, group 1 protein	108	281637-79_2_ORF2 (+1)	17	Q39138	2.65719E-05	−2.52	20	5
Embryo defective 1579 (emb1579)	1,082	27243-483_2_ORF1 (+1)	123	F4IS91	9.02076E-73	−1.77	20	14
Late embryogenesis abundant (LEA) protein in group 3	61	253001-98_1_ORF1	14	Q9SKP0	1.43925E-11	−1.55	49	9
Villin-2; Ca(2+)-regulated actin-binding protein	1,014	80596-281_5_ORF2	92	O81644	0	−2.58	3.2	3
NAP1-related protein 2	410	354891-42_3_ORF1 (+1)	33	Q8LC68	4.8665E-99	−1	7.8	2
Persulfide dioxygenase ETHE1 homolog	399	249504-100_3_ORF2	32	Q9C8L4	9.9249E-145	−2.32	12	3
ATP-dependent zinc metalloprotease FTSH 2	956	64641-315_3_ORF2	75	A0A1P8AXC1	0	−1.32	5	4
UPF0603 protein	396	128797-216_2_ORF2	32	Q9ZVL6	4.7595E-112	−1	13	3
Arginase/deacetylase superfamily protein	584	1738-1468_5_ORF1 [4]	43	Q9ZPF5	6.0531E-168	−4.46	33	10
NAD(P)-binding Rossmann-fold superfamily protein	296	66706-309_6_ORF2	28	Q94EG6	7.7668E-135	−1.13	25	6
GroES-like zinc-binding dehydrogenase family protein	583	70975-299_4_ORF2	42	Q96533	0	−1.32	9.4	3
Nudix hydrolase homolog 8	428	416362-16_5_ORF3 (+3)	34	Q8L7W2	2.86086E-92	−2.32	8.9	3
Flavone 3'-O-methyltransferase 1	528	1607-1505_6_ORF1	39	Q9FK25	1.09086E-60	−2.58	8.7	4
Glutamate decarboxylase 1	797	tr|B9H3K1|B9H3K1_POPTR	56	Q42521	0	−1.58	5.7	5
Glutathione S-transferase TAU 20	50	423746-11_3_ORF1 [2]	14	Q8L7C9	2.22255E-16	−1	23	4
AMP-dependent synthetase and ligase family protein	814	681-1862_1_ORF2	58	Q9SMT7	0	−1	4.3	2
Phosphoserine aminotransferase 1, chloroplastic	647	350227-43_2_ORF2	46	Q96255	0	−1.74	12	4
Embryo defective 2171 (emb2171)	76	18818-572_2_ORF2 (+3)	16	P49690	1.51032E-96	−1.32	17	3
RNA-binding (RRM/RBD/RNP motifs) family protein (LIF2)	873	71037-299_5_ORF2	63	Q9ASP6	1.00527E-36	−1.32	7.2	4
Sec14p-like phosphatidylinositol transfer family protein	922	420882-13_1_ORF2	68	Q94C59	5.0715E-123	−2.66	15	10
Rubber elongation factor protein (REF)	194	sp|O82803|SRPP_HEVBR	22	Q9MA63	1.01539E-67	−3.17	15	3
Encodes a dual-targeted protein believed to act as a pyruvate, orthophosphate dikinase	1042	418542-15_2_ORF1 [22]	102	O23404	0	−1.64	12	10
Peroxidase family protein	476	167942-169_1_ORF2	36	Q9LSP0	8.46546E-91	−2.58	14	4
Mediator of RNA polymerase II transcription subunit 36a	324	6971-870_2_ORF2 [4]	29	Q94AH9	2.3256E-162	−1.91	24	6
Pollen Ole e 1 allergen and extensin family protein	471	133239-212_4_ORF2 (+1)	36	Q8RWG5	6.10012E-29	−1.74	11	4
Pectin lyase-like superfamily protein	555	18361-579_1_ORF2 (+2)	41	Q8VYZ3	3.1298E-116	−1	11	3

### Quantification of Endogenous Phytohormones

The endogenous levels of the plant growth regulators analyzed are shown in Table 3. The auxin IAA was found in a greater quantity in *D. oreades* gametophytes than in any growth stage of *D. affinis*, and in spatulate compared to filamentous and cordate gametophytes. Regarding cytokinins, BA peaked in the filamentous gametophytes of *D. affinis*, and Z and ZR showed low levels in *D. affinis* (<0.1 ng/g FW) and markedly higher in the sexual counterpart.

**Table 3:** Endogenous content of phytohormones in three growth stages of apogamous fern *Dryopteris affinis* and one stage of its relative *D. oreades*.

	***Dryopteris affinis*** **ssp**. ***affinis***						***D. oreades***
	**Filamentous**		**Spatulate**		**Heart**		**Heart**
**ng/gFW**	**Mean**	**SE**	**Mean**	**SE**	**Mean**	**SE**	**Mean**
IAA	0.33	0.02	0.16	0.03	0.29	0.05	0.88
BA	0.31	0.03	0.18	0.02	0.18	0.02	0.19
Z	0.02	0.00	0.03	0.00	0.02	0.00	0.16
ZR	0.98	0.08	0.94	0.07	1.00	0.10	3.13
DHZ	0.03	0.00	0.03	0.00	0.02	0.00	0.02
DHZR	0.16	0.02	0.11	0.01	0.22	0.04	0.08
iP	0.23	0.01	0.24	0.01	0.25	0.01	0.25
iPR	1.19	0.09	0.70	0.09	0.83	0.06	2.29
GA3	0.74	nd	3.61	0.81	1.33	0.13	2.33
GA4	141.56	35.14	135.60	19.48	164.57	21.26	278.73
ABA	2.89	0.20	2.21	0.06	2.17	0.13	3.48
SA	35.53	5.77	20.27	0.85	14.70	0.18	8.42
CS	nd	nd	nd	nd	10.13	1.75	369.92
BL	2.55	0.67	4.53	0.54	1.58	0.58	58.64

*Data are mean ± standard error. IAA,indol-3-acetic acid, BA, 6-benzylaminopurine; Z, trans-Zeatin; ZR, Zeatin riboside; DHZ, dihydrozeatin; DHZR, dihydrozeatin riboside; iP, isopentenyl adenine; iPR, isopentenyl adenosine; ABA,abscisic acid; SA, salicylic acid; GA_3_, gibberellic acid; GA_4_, gibberellin 4; BL, brassinolide; CS, castasterone; nd, below detection limit*.

No differences were found in the content of DHZ, while the levels of its riboside, DHZR, significantly shrank in spatulate gametophytes of *D. affinis* compared to cordate gametophytes, dropping considerably in the sexual parent. Finally, no differences were found regarding the isoprenoid cytokinin iP, while the levels of its riboside, iPR, was lower in the spatulate and cordate than in the filamentous apogamous gametophytes, and also than in the sexual gametophytes.

The levels of plant growth inhibitor ABA increased in the sexual gametophytes and no differences were observed among the three developmental stages of apogamous gametophytes. Salicylic acid showed substantial differences among the four gametophyte samples. Levels up to 35 ng/g FW were detected in filamentous apogamous gametophytes, falling significantly in sexual ones. In relation to the tested gibberellins, high levels of GA_4_ were noticed in sexual gametophytes, being also significant in apogamous ones. In addition, GA_3_ reached a maximum in spatulate apogamous gametophytes. Finally, on one hand, the brassinosteroid CS was undetected from filamentous and spatulate apogamous samples, and low in cordate apogamous gametophytes. On the other hand, this phytohormone was 10 times more abundant in sexual than in apogamous gametophytes. BL is 25 times more abundant in sexual than in apogamous gametophytes (but only one sample was achieved). In addition, significant differences were found between spatulate and the other apogamous stages.

**Figure 6:**
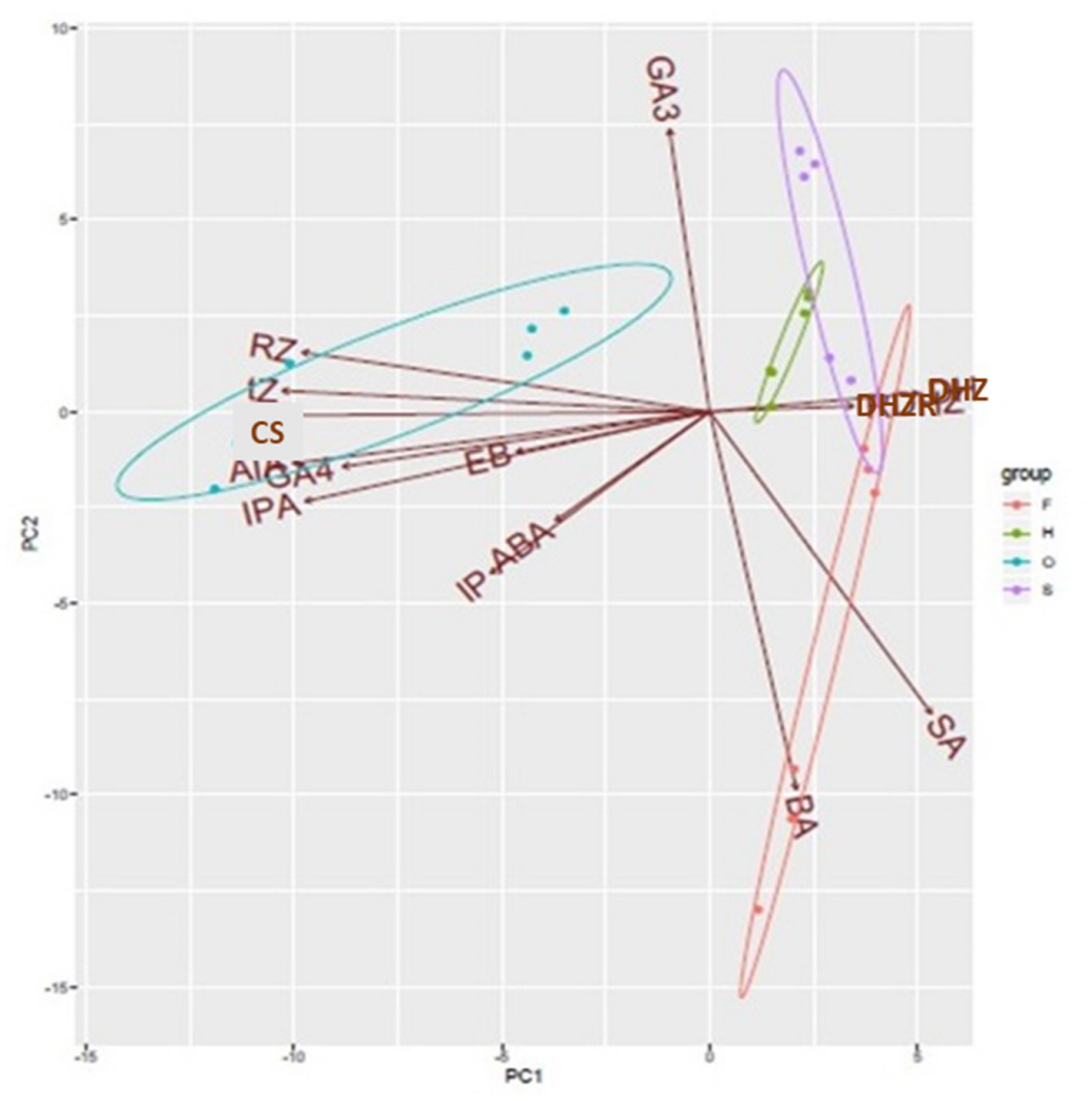
PCA biplot showing the contribution of all variables to the variation in the endogenous content of phytohormones in filamentous (F), spatulate (S), and heart-shaped (H) gametophytes of the apogamous species *Dryopteris affinis*, and heart-shaped gametophytes (O) of its sexual relative *D. oreades*. Ellipses encircle group samples from the same origin. IAA, indol-3-acetic acid; BA, 6-benzylaminopurine; Z, trans-Zeatin; Zr, Zeatin riboside; DHZ, dihydrozeatin; DHZR, dihydrozeatin riboside; iP, isopentenyl adenine; iPR, isopentenyl adenosine; ABA, abscisic acid; SA, salicylic acid; GA_3_, gibberellic acid; GA_4_, gibberellin 4; BL, brassinolide; CS, castasterone.

Principal Component Analyses (PCA) revealed that one component (PC1) included the most part of the variance (90%), splitting sexual and apogamous gametophytes (Figure 6). Biplot shows the phytohormones grouping into the four gametophyte samples. DHZ and DHZR are common to filamentous and spatulate *D. affinis* gametophytes, and GA_3_ is the hormone which differentiates both groups of samples. Apogamous filamentous samples showed variation with a great dispersion, and they shared differences with both spatulate and cordate stages by the cytokinin BA and salicylic acid.

## Discussion

Our results revealed substantial differences in the protein content between apogamous and sexual gametophytes of two closely related woodfern species. Specifically, *D. affinis* and *D. oreades* showed differences in terms of the expression of proteins involved in some biological, molecular and cellular aspects of plant development. Part of these differences can be explained by the distinct reproductive systems. Furthermore, although both species share the *D. oreades* genome, *D. affinis* has an additional genome (Fraser-Jenkins, 1980) whose expression also contributes to the divergent proteomic profiles.

### Apogamous Gametophytes Upregulate Proteins Related to Protein Folding, Transport, Targeting, Proteolysis, Ribosome Biogenesis, Gene Expression and Translation

It has been reported an important role of protein metabolism on apomixis in angiosperms (Schmidt, 2020). Certainly, cellular processes related to proteins, such as in transport, folding, targeting or proteolysis are upregulated in the gametophyte of our apogamous species. For instance, we found RAN3, a small GTP-binding nuclear protein, required for the import of protein into the nucleus, RNA export, chromatin condensation and the control of cell cycle (Yano et al., 2006); and a puromycin-sensitive aminopeptidase, which plays an essential role during prophase I of meiosis for correct meiotic recombination in both male and female gametophytes (Sánchez-Morán et al., 2004). In addition, protein folding chaperones such as HSP60, HSP60-2, and the translation initiation factors FUG1, IF3-4 or ELF 3C, were found. Then, two signal chloroplastic recognition particles: cpSRP43 (CAO) and cpSRP54, involved in protein heterotrimerization, deserve to be mentioned. They act as a highly specific chaperones for LHCPs, preventing aggregation and being able to dissolve aggregates (Falk and Sinning, 2010; Falk et al., 2010). G-protein signaling is an integral part of the G-protein network important in many agronomic traits, including architecture and grain yield, and which is lost in many monocots (Bhatnagar and Pandey, 2020). In *D. affinis*, another interesting annotation is the chaperone protein htpG family protein or SHD, which encodes an ortholog of GRP94, an ER-resident HSP90-like protein involved in regulation of meristem size and organization. This protein is suggested to be required for the correct folding and/or complex formation of CLV proteins (Ishiguro et al., 2002), and it is also involved in resistance to tunicamycin- or high calcium-induced ER stress (Chong et al., 2015). Indeed, Hsp70s are key components that facilitate folding of *de novo* synthesized proteins, assist translocation of precursor proteins into organelles, and are responsible for degradation of damaged proteins under stress conditions (Lee et al., 2009).

In apogamous gametopytes, we also found proteins related to the ribosome organelle, including a wide number of processes such as ribosome biogenesis, processing, gene expression, translation and cell cycle control. As an example, the protein CPR, member of metallopeptidase M24 family, is involved in the regulation of rRNA processing and ribosome assembly, and is required for expression of cell cycle genes such as CYCD3-1, RNR2A, and CDKB1-1, playing a role in the entry into mitosis. Besides, it promotes, in a dose- and auxin-dependent manner, organ growth by stimulating both cell proliferation and expansion, via the regulation of RBR1 levels (Horváth et al., 2006). The list also included an emb3010 or 40S ribosomal protein S6-2, which may play an important role in controlling cell growth and proliferation through the selective translation of particular classes of mRNAs (Zhou et al., 2015).

Ribosome assembly and function are correlated with the activity of RNA binding proteins, often associated to ribonucleoprotein complexes of RNA as well as helicases (Schmidt, 2020). The former includes proteins like the phosphoprotein AtLa1, required for normal ribosome biogenesis and embryogenesis (Fleurdépine et al., 2007), and the latter, a 50S ribosomal protein L13 or embryo defective 1473, involved in translation and biological processes such as embryo development ending in seed dormancy or stress tolerance (Moin et al., 2016).

RNA helicases are considered of crucial importance for gene regulation of developmental processes, exerting an epigenetic control. In the apogamous species, two helicases, to unwind nucleic acids, were reported: the emb1138, member of the DEAD-box ATP–dependent RNA helicase 3, and At3G62310, a probable pre-mRNA-splicing factor ATP-dependent RNA helicase DEAH2, which might be involved in pre-mRNA splicing. The DEAD-box helicases are involved in various aspects of RNA metabolism, including nuclear transcription, pre-mRNA splicing, ribosome biogenesis, nucleocytoplasmic transport, translation, RNA decay and organellar gene expression. Different studies provided evidence of RNA helicases to be likely involved in regulating apomictic development (Schmidt, 2020). In *A. thaliana*, mutants for the RNA helicase MNEME form unreduced gametophytes, resembling apospory (Schmidt et al., 2011). Also, in apomictic *Brachiaria brizantha* and in *Hypericum perforatum*, BrizHELIC and a of MATERNAL EFFECT EMBYO ARREST29, are differentially expressed in tissues of apomictic plants (Silveira et al., 2012; Barcaccia and Albertini, 2013). In our work, a homologue of MATERNAL EFFECT EMBRYO ARREST 59 (MEE59) is up regulated in the apogamous gametophyte, but its biological function is unknown. Furthermore, we found TFL2, a chromo domain-containing protein LHP1, structural component of heterochromatin, and the transducin/WD40 repeat-like superfamily protein, which is a component of the PAF1 complex (PAF1C), involved in epigenetic gene repression of several floral homeotic genes, such as FLT, that regulates flowering time, and is required for maintenance of vernalization-induced repression of FLC (Kotake et al., 2003; Zhang et al., 2003; Xu and Shen, 2008). An interesting finding is the ortologue of the transcriptional regulation protein Methyl-CpG-binding domain-containing protein 10, required for nucleolar dominance that leads to the silencing of rRNA genes inherited from one progenitor in interspecific hybrids (Preuss et al., 2008). It must be noted that *D. affinis* ssp. *affinis* is actually a fertile hybrid in which both sporophyte and gametophyte have two genomes from two different species (Fraser-Jenkins, 1980). Finally, it is reported the mitochondrial pentatricopeptide repeat-containing protein At1g63130, a trans-acting siRNA (ta-siRNA) generating locus (Yoshikawa et al., 2005).

Some annotated proteins may be involved on reproduction, such as D-3-phosphoglycerate dehydrogenase 1, which apart from participating in the plastidial phosphorylated pathway of serine biosynthesis (PPSB) (Benstein et al., 2013), might act on pollen development, megagametogenesis or seed dormancy (Toujani et al., 2013). Also, a DC1 domain-containing protein, associated to the generation of lipid signaling molecules in pistil (Qin et al., 2009), a dihydrolipoyl dehydrogenase 1, highly expressed in developing seeds, and the translation elongation factor emb2726, member of the broad mutant family found in *Arabidopsis*, EMBRYO DEFECTIVE (Meinke, 2020).

Our results give support to a role of the metabolism of arginin, precursor of polyamines, on gametophyte reproduction, due to the increase of the protein argininosuccinate synthase and carbamoyl-phosphate synthase. A possible role of polyamines on gametophyte development is a recurring data in our previous analyses in these fern species (Grossmann et al., 2017; Wyder et al., 2020). Finally, the metabolism of cell wall pectins acquires some importance with the protein UGD2, which is involved in the biosynthesis of UDP-glucuronic acid (UDP-GlcA), providing nucleotide sugars for cell-wall polymers. A possible connection to the apogamy will need further research.

### Sexual Gametophytes Upregulate Proteins Related to Starch and Sucrose Metabolism, Generation of Precursor Metabolites and Energy, and Photosynthesis

Starch and sucrose metabolism appear to be relevant processes in the sexual gametophytes. Specifically, among other proteins upregulated in the studied female gametophytes, we found alpha and beta amylase, pfkB-like carbohydrate kinase family protein, phosphoglucan, water dikinase (PWD), cytosolic fructose 1-6-bisphosphatase, a probable sucrose-phosphate synthase 3, glucose-1-phosphate adenylyltransferase, chloroplastic glucose-6-phosphate isomerase 1, etc. In *Ceratopteris thalictroides*, proteins linked to photosynthesis were over-represented in hermaphrodite gametophytes compared to male ones induced by antheridiogens, which had smaller size (Chen et al., 2019).

Regarding the generation of precursor metabolites and energy annotations, several proteins of both aerobic or anaerobic paths, such as the citrate cycle (TCA cycle), oxidative phosporilation or glucolysis/gluconeogenesis, significantly increased in sexual gametophytes. Moreover, in these gametophytes the number of proteins upregulated in photosynthesis and antenna proteins was double that of the apogamous one, and they act on chromophore-linkage and assembly and repair of the photosystetic apparatus (PSBB, PSAB, LHCB5, LHCB3, LHCA3, VAR2, PSB27, TLP18.3, PORA, PORC). PORA, for instance, may function as a photoprotectant during the transitory stage from dark to light, and also in photomorphogenesis and throughout the plant life under specific light conditions (Paddock et al., 2012). There are also proteins associated with pigment biosynthetic processes, such as the magnesium-chelatases ALB1 and GUN5, which can have other functions as GUN5 acting in ABA signaling (Du S. Y. et al., 2012).

In our experiments, both sexual and apogamous gametophytes were cultured under similar conditions of light, photoperiod, humidity, nutrients, or pH. Although it was not scored, the growth rate of gametophytes was clearly slower in *D. oreades* than in *D. affinis*. However, final size of the sexual gametophyte was bigger than that of the apogamous one. In the gametophytes, although scarce, there are some data supporting differences in photochemical efficiency linked to sex gender, increasing in female individuals, which thus could provide more energy for the development of archegonia, fertilization and the further growth of sporophytes (Valledor et al., 2014; Slate et al., 2017; Chen et al., 2019). Our data suggest that the sexual gametophytes are more energy-demanding than the apogamous counterparts. We propose that the gametophyte of *D. affinis* devotes all resources to quickly grow and differentiate an embryo, while the female gametophytes have to attain a more complex organization level, building a decisive central area, the archegonium cushion, a several cell layers thick which is the place where the archegonia and future embryo will develop. At first glance, the larger and more complex sexual gametophyte could thus demand more energy than the smaller and fast-growing apogamous gametophytes.

The lipid metabolism seems especially important in the sexual gametophytes, upregulating proteins involved in linoleic acid metabolism such as the lipoxygenases LOX1 and LOX 3, as well as the peroxisomal fatty acid beta-oxidation multifunctional protein MFP2. These proteins are linked to functions such as growth and development, pest resistance, senescence or responses to wounding (Vellosillo et al., 2007), and in the tree fern *C. delgadii*, key enzymes in fatty acid biosynthesis were told to have an important role during embryogenesis (Domzalska et al., 2017).

Other proteins found in the sexual gametophyte are related to different aspects of reproduction, such as embryo defective 1579, involved in embryo development ending in seed dormancy, several LEA proteins such as LEA4-1 or ECP63, which may be linked to the BHLH109-mediated regulation of somatic embryogenesis (Nowak and Gaj, 2016), and Villin-2, a Ca(2+)-a regulated actin-binding protein, required for the construction of actin collars in pollen tubes (van der Honing et al., 2012; Qu et al., 2013). In addition, NAP1-related protein 2 acts as histone H2A/H2B chaperone in nucleosome assembly, being essential, together NRP1, for the maintenance of cell proliferation and differentiation in postembryonic root growth, and for intramolecular and intermolecular somatic homologous recombination (Zhu et al., 2006). Furthermore, we annotated two interesting proteins: an ADP-ribosylation factor-like protein, member of ARF GTPase family partaking in cell division, expansion, and cellulose production; and the monocopper oxidase-like protein SKU5, a GPI-anchor protein involved in directed root tip growth (Sedbrook et al., 2002).

Other interesting protein is a flavone 3'-Omethyltransferase, involved in melatonin biosynthesis (Byeon et al., 2014), and the protein glutamate decarboxylase 1, which catalysis the production of GABA (Bouché et al., 2004). Finally, it deserves to mention the protein FIB2, a S-adenosyl-L-methionine-dependent methyltransferase, which has been speculated to be involved on methylation of RNAs and proteins, including histones, having an important role on defense against bacterial pathogens (Seo et al., 2019).

### Phytohormones: Hormonal Content and Protein Regulation

Information acquired so far about the physiology and molecular events operating inside this free-leaving fern generation is scarce. As far as we know, the present study represents the first assessment of a large number of phytohormones in fern gametophytes, and shows changes in phytohormone profiles related to reproductive systems in non-model species.

The presence of SA at higher levels in the filamentous apogamous gametophytes could be related to a stressful environment as could be *in vitro* culture itself, especially at the beginning of gametophyte development (Kosakivska et al., 2019; Wyder et al., 2020). SA influences a wide range of processes, including seedling establishment and responses to abiotic and biotic stresses (Vlot et al., 2009), being one of the first features in plant-microbe interactions that is present in basal-branching algae (Pieterse et al., 2012; Hori et al., 2014). Although this phytohormone showed higher levels at the filamentous stage, the proteomic data point out an important presence of its metabolic pathways along the whole gametophyte development, as shown later.

Although very simple morphologically, gametophytes go through successive developmental changes until sexual maturity. An early crucial event in the formation of the two-dimensional gametophyte involves the re-orientation of cell plate, from a transverse to a longitudinal alignment (Nayar and Kaur, 1971; Banks, 1999; Racusen, 2002). Our results suggest a possible connection between the cytokinin BA and the filamentous morphology, and between GA_3_ and the spatulate shape. Cytokinins might affect the rate and pattern of cell division, cell elongation and cell differentiation in ferns (Romanov, 2009). Although gametophytes of *B. spicant* and *Osmunda regalis* became shorter, widened and lacking meristem in response to cytokinins (Menéndez et al., 2006a; Greer et al., 2012), these phytohormones induced transition from one- to two-dimensional growth in *C. richardii* (Spiro et al., 2004). The increase of GA_3_ in our spatulate gametophytes is consistent with previous data with other sexual and apogamous fern species such as *B. spicant, Anemia phillitidis*, and *Polystichum aculeatum* (Kazmierczak, 2003; Menéndez et al., 2006b,c, 2009; Kosakivska et al., 2020).

The levels of IAA, Z, ZR, iPR, the gibberellin GA_4_, the brassinosteoid castasterone and ABA were higher in sexual cordate gametophytes than in the apogamous cordate ones. The primary role of zeatin riboside in reproductive processes has been stated in ferns (Abul et al., 2010; Vedenecheva and Sytnik, 2013; Vedenicheva and Kosakivska, 2016, 2018). In the last case, zeatin and seatin riboside levels increased in the sporophyte of the fern *D. filix-mas* when sporulation happens, while zeatin and zeatin riboside content became higher during intensive vegetative growth in *P. acculeatum*. Recently, an increase of IAA, ZR, iP, iPR, GA_3_ and ABA in the cordate gametophyte of *P. aculeatum* has been documented by the same authors (Kosakivska et al., 2020), pointing out a key role of these phytohormones in the regulation of growth and development of the cushion. The iP bases cytokinins are characteristic of mosses and ferns, being more difficult to be detected zeatin derivatives (Johri, 2008). Additionally, in cordate gametophytes of *D. filix-mas* an increase in the IAA and Z, at the formation of both sexual organs, archegonia and antheridia, has been reported (Kosakivska et al., 2019). These authors concluded also that GA_3_ was dominant at all stages of gametophyte development, reaching the highest content during the development of the sexual organs. The gibberellin GA_4_ prevails over GA_3_ either in apogamic or sexual development, being especially high in the latter. Previously, high levels of GA_4_ and GA_7_ were assessed in *D. affinis*, as an apogamous embryo evolved (Menéndez et al., 2006c), and in gametophytes governed by an antheridiogen system, GA_4_ has been associated with the induction of antheridia, and also with contributing to the genetic exchange (Tanaka et al., 2014). The accumulation of GA_4_ in the cordate sexual gametophytes of *D. oreades* suggests a wider role on gametophyte development not yet dilucidated by the gibberellins.

The sexual gametophytes also had a noticeable increase in ABA. It has recently been reported that the origin of the core ABA signaling pathway in seed plants might lie in the sexual differentiation of ferns (McAdam et al., 2016). In female gametophytes of *D. oreades*, a possible connection of ABA with their antheridiogen-release capacity could be to protect themselves against the activity of these pheromone, preserving the female condition (Hickok, 1983; Banks et al., 1993). Regarding the active brassinosteroids analyzed, both BL and CS were detected. Many processes have been linked with brassinosteroids function such as cell elongation, cell division, reproductive and vascular development, stress responses, or senescence, but information about their role on ferns is scarce (Gómez-Garay et al., 2018). Interestingly, the amount of CS was high, pointing at some possible role of brassinosteroids on sexual development of gametophytes in *D. oreades*. Moreover, CS was identified in most of the ferns studied by Yokota et al. (2017) but not BL.

As noted above, our proteomic analysis revealed a myriad of proteins related to the biosynthesis and/or function of the analyzed phytohormones, giving strong support to the important role of these regulators on gametophyte development. In the apogamous gametophyte, the histidine phosphotransfer protein AHP1, linked to cytokinin signaling was annotated (Hwang et al., 2002), and the protein, emb22 (also named Gurke or Pasticcino3), which is an acetyl-CoA carboxylase 1, related to cell proliferation and tissue patterning (Baud et al., 2003), embryo morphogenesis (Torres-Ruiz et al., 1996); or supressing cytokinin activity (Faure et al., 1998). Also the protein Cand1, required for SCF^TIR^ activity, participating in several resposes such as response to auxin (Cheng et al., 2004). In addition, there are proteins on defense or stress, such as prohibitin-3, required to regulate ethylene-mediated signaling, and nitric oxide (NO)-mediated responses (Van Aken et al., 2007; Wang et al., 2010).

On the other hand, sexual gametophytes overexpressed the plasma membrane-type ATPase 1, which has been suggested to be involved in brassinosteroid signaling (Ladwig et al., 2015); tryptophan synthase beta type 2 (TSBtype2), acting on the auxin biosynthesis (Zhao, 2014); proteins linked to the ABA response, such as those of NAD(P)-binding Rossmann-fold superfamily proteins (Ghelis et al., 2008); to polyamines or proline as arginase/deacetylase (Patel et al., 2017), whose over-expression decreases susceptibility to the fungal pathogen *Botrytis cinerea* (Brauc et al., 2012). We also found Nudix hydrolase homolog 8, which may act on SA signaling (Fonseca and Dong, 2014).

Another interesting protein is a transcriptional activator belonging to RNA-binding (RRM/RBD/RNP motifs) family protein, antagonist and promoter of polycomb LHP1 gene regulation activity, to regulate the transcription of stress-responsive and flowering genes (Latrasse et al., 2011). Besides, it may function as a suppressor of cell-autonomous immune responses involving glucosinolates, SA, and JA pathways toward pathogenic bacteria and fungi (Le Roux et al., 2014). Finally, glutathione S-transferase TAU 20 is another protein upregulated in the sexual gametophyte, involved in the regulation of far-red light influence on development as a regulator of the interplay between light and JA signaling (Chen et al., 2007, 2017) and playing a role in gravitropic signal transduction (Schenck et al., 2013).

### Conclusions

Qualitative and quantitative differences in protein and phytohormone profiles between apogamous and sexual gametophytes are reported. Our results indicate that phytohormone contents vary either between cordate apogamous gametophytes and their sexual counterpart. Our main conclusions were: (1) Seven out of fourteen phytohormones accumulated more in the sexual (female) gametophyte, especially auxin IAA, the cytokinins Z, ZR, iPR, the active gibberellin GA_4_, and the active brassinosteoids CS. (2) The proteins upregulated in the apogamous species are associated with the primary metabolism of aminoacids, peptides and proteins (including folding, transport and proteolysis), nucleic acids and cofactors, nitrogen metabolism, ribosome biogenesis, translation and gene expression and stress response. (3) The sexual counterpart accumulated more proteins coping with starch and sucrose metabolism, generation of energy and photosynthesis, lipid oxidation and response to hormones. Asexual vs. sexual gametophyte involves two different metabolic scenarios, with apogamous reproduction more connected to stress responses while sexual reproduction is more resource demanding.

## Data Availability Statement

The datasets presented in this study can be found in online repositories. The names of the repository/repositories and accession number(s) can be found in the article/Supplementary Material.

## Author Contributions

HF and UG: conceive the project. HF, AR, and MC: *in vitro* culture of ferns and sampling. LQ: supplied plant material. AR and VQ: statistical analyses. JG: performed bioinformatics. VG: protein extraction and purification. IF and LR: phytohormones analyses. All authors discussed the results and contributed to the final manuscript.

## Funding

This work was supported by the University of Zurich and Project PRIME-XS-0002520 funded by the European Union's 7th Framework Program.

## Conflict of Interest

The authors declare that the research was conducted in the absence of any commercial or financial relationships that could be construed as a potential conflict of interest.

## Publisher's Note

All claims expressed in this article are solely those of the authors and do not necessarily represent those of their affiliated organizations, or those of the publisher, the editors and the reviewers. Any product that may be evaluated in this article, or claim that may be made by its manufacturer, is not guaranteed or endorsed by the publisher.

## Abbreviations

ABA: abscisic acid
BA: 6-benzylaminopurine
BL: brassinolide
CS: castasterone
DA: *Dryopteris affinis*
DO: *Dryopteris oreades*
DHZ: dihydrozeatin
DHZR: dihydrozeatin riboside
GA_3_: gibberellic acid
GA_4_: gibberellin GA4
IAA: indole-3-acetic acid
iP: isopentenyl adenine
iPR: isopentenyl adenosine
JA: jasmonic acid
MS/MS: mass spectrometry
MS: Murashige and Skood mineral medium (1962)
SA: salicylic acid
ZR: trans-zeatin riboside
Z: trans-zeatin.
